# A Novel Marsupial Hepatitis A Virus Corroborates Complex Evolutionary Patterns Shaping the Genus Hepatovirus

**DOI:** 10.1128/JVI.00082-18

**Published:** 2018-06-13

**Authors:** Ianei de Oliveira Carneiro, Anna-Lena Sander, Namá Silva, Andres Moreira-Soto, Andrea Normann, Bertram Flehmig, Alexander N. Lukashev, Andreas Dotzauer, Nicolas Wieseke, Carlos Roberto Franke, Jan Felix Drexler

**Affiliations:** a Federal University of Bahia, Salvador, Brazil; b Charité-Universitätsmedizin Berlin, corporate member of Freie Universität Berlin, Humboldt-Universität zu Berlin, and Berlin Institute of Health, Institute of Virology, Berlin, Germany; c Mediagnost, Reutlingen, Germany; d Martsinovsky Institute of Medical Parasitology, Tropical and Vector Borne Diseases, Sechenov University, Moscow, Russia; e Chumakov Federal Scientific Center for Research and Development of Immune and Biological Preparations, Russian Academy of Sciences, Moscow, Russia; f Laboratory of Virus Research, University of Bremen, Bremen, Germany; g Swarm Intelligence and Complex Systems Group, Department of Computer Science, Leipzig University, Leipzig, Germany; h German Centre for Infection Research‡; University of Southern California

**Keywords:** hepatitis A virus, small mammals, evolution, pathogenesis

## Abstract

The discovery of highly diverse nonprimate hepatoviruses illuminated the evolutionary origins of hepatitis A virus (HAV) ancestors in mammals other than primates. Marsupials are ancient mammals that diverged from other Eutheria during the Jurassic. Viruses from marsupials may thus provide important insight into virus evolution. To investigate Hepatovirus macroevolutionary patterns, we sampled 112 opossums in northeastern Brazil. A novel marsupial HAV (MHAV) in the Brazilian common opossum (Didelphis aurita) was detected by nested reverse transcription-PCR (RT-PCR). MHAV concentration in the liver was high, at 2.5 × 10^9^ RNA copies/g, and at least 300-fold higher than those in other solid organs, suggesting hepatotropism. Hepatovirus seroprevalence in D. aurita was 26.6% as determined using an enzyme-linked immunosorbent assay (ELISA). Endpoint titers in confirmatory immunofluorescence assays were high, and marsupial antibodies colocalized with anti-HAV control sera, suggesting specificity of serological detection and considerable antigenic relatedness between HAV and MHAV. MHAV showed all genomic hallmarks defining hepatoviruses, including late-domain motifs likely involved in quasi-envelope acquisition, a predicted C-terminal pX extension of VP1, strong avoidance of CpG dinucleotides, and a type 3 internal ribosomal entry site. Translated polyprotein gene sequence distances of at least 23.7% from other hepatoviruses suggested that MHAV represents a novel Hepatovirus species. Conserved predicted cleavage sites suggested similarities in polyprotein processing between HAV and MHAV. MHAV was nested within rodent hepatoviruses in phylogenetic reconstructions, suggesting an ancestral hepatovirus host switch from rodents into marsupials. Cophylogenetic reconciliations of host and hepatovirus phylogenies confirmed that host-independent macroevolutionary patterns shaped the phylogenetic relationships of extant hepatoviruses. Although marsupials are synanthropic and consumed as wild game in Brazil, HAV community protective immunity may limit the zoonotic potential of MHAV.

**IMPORTANCE** Hepatitis A virus (HAV) is a ubiquitous cause of acute hepatitis in humans. Recent findings revealed the evolutionary origins of HAV and the genus Hepatovirus defined by HAV in mammals other than primates in general and in small mammals in particular. The factors shaping the genealogy of extant hepatoviruses are unclear. We sampled marsupials, one of the most ancient mammalian lineages, and identified a novel marsupial HAV (MHAV). The novel MHAV shared specific features with HAV, including hepatotropism, antigenicity, genome structure, and a common ancestor in phylogenetic reconstructions. Coevolutionary analyses revealed that host-independent evolutionary patterns contributed most to the current phylogeny of hepatoviruses and that MHAV was the most drastic example of a cross-order host switch of any hepatovirus observed so far. The divergence of marsupials from other mammals offers unique opportunities to investigate HAV species barriers and whether mechanisms of HAV immune control are evolutionarily conserved.

## INTRODUCTION

Hepatitis A virus (HAV) is a ubiquitous cause of acute viral hepatitis in humans, causing about 11,000 deaths worldwide per year (1). HAV belongs to the genus Hepatovirus within the family Picornaviridae (2). HAV stands out from other picornaviruses in its ability to occur as typical nonenveloped viruses in feces and as lipid-layered particles in blood (3). Additionally, the unique structural properties of HAV, resembling those found in ancestral insect viruses, suggest that HAV is an ancient picornavirus (4).

HAV was long thought to be restricted to primates, with genotypes I to III found in humans and genotypes IV to VI, termed simian HAV (SHAV), found in nonhuman primates (2). Because HAV engenders long-lasting immunity following infection, how the virus may have survived in scattered prehistoric human populations has long been enigmatic (5). Only recently, highly diverse nonprimate hepatoviruses were discovered, suggesting that HAV ancestors may have evolved in mammals other than primates prior to their introduction into humans (5).

**FIG 1:**
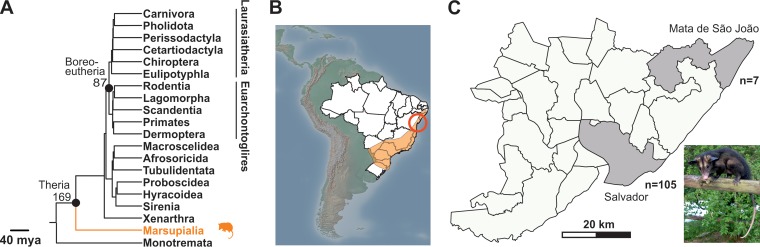
Phylogeny of hepatovirus hosts and sampling sites. (A) Mammalian phylogeny showing the time of divergence between marsupials and therians, including a monotreme outgroup, according to data from reference 11. mya, million years ago. (B) South America, Brazil, and sampling site in Bahia (red circle). The orange area shows the geographic range of Didelphis aurita, the most frequently sampled marsupial in this study. The distribution was retrieved from the IUCN Red List of Threatened Species (https://doi.org/10.2305/IUCN.UK.2015-4.RLTS.T40500A22175929.en) on 2 January 2018 and mapped using QGIS (www.qgis.org) and open source data from Natural Earth (http://www.naturalearthdata.com). (C) Total samples obtained per site. Inset, image of D. aurita (copyright, Pedro Lima; reproduced with permission).

The expanded genus Hepatovirus now includes at least 16 putative virus species (6). The majority of novel hepatoviruses were obtained from bats and rodents (5, 7). The corresponding host orders Chiroptera and Rodentia are the most speciose among mammals, and both are major sources of novel viruses (8, 9). Other mammalian orders carrying hepatoviruses include Primates, Scandentia, Eulipotyphla, and Carnivora (6, 10). These orders all belong to a clade of placental mammals termed Boreoeutheria (6) (Fig. 1A).

Boreoeutherian orders diversified about 87 million years ago (mya), during the upper Cretaceous, and their phylogenetic relationships are not easily reconciled (11). The rapid diversification that occurred at the root of known hepatovirus hosts challenges coevolutionary assessments at ancestral nodes of the Hepatovirus phylogeny. Additionally, host genetic relatedness facilitates cross-host infections of pathogens (12). Spillover infections between genetically related hosts are thus not easily differentiated from coevolutionary relationships.

The Hepatovirus genealogy is likely complex. In preliminary work, we noted conflicting phylogenies of hosts and nonprimate hepatoviruses that suggested several nonrecent hepatovirus host switches (5). In follow-up analyses, we showed that recombination events involving highly diverse hepatoviruses from different mammalian orders corroborated these host switches (6). On the other hand, we showed that surprisingly speciose monophyletic clades of bat hepatoviruses exist across different continents, which may hint at still-undefined long-term evolutionary relationships (6).

Comparative phylogenomic analyses of hepatoviruses from highly divergent hosts may provide important insights into the macroevolutionary patterns of this ancient viral genus. The order Marsupialia is one of the oldest mammalian orders and diverged from the ancestor of Boreoeutheria about 170 mya (11) (Fig. 1A). Marsupials such as opossums likely originated from South American ancestors and nowadays are endemic to Australasia and the Americas only (13).

To further elucidate Hepatovirus macroevolutionary patterns, we investigated opossums from Brazil for hepatoviruses by using molecular, serological, and bioinformatic tools.

## RESULTS

### Fieldwork.

During July and August 2015, we sampled opossums in remaining Atlantic rain forest patches in the northeastern Brazilian state of Bahia, including the capital (Salvador) and a municipality termed Mata de São João, located 53 km away (Fig. 1B and C). For 55 animals captured alive, only a serum specimen was taken. Another 57 animals were obtained dead from the wildlife clinic of the Department for Veterinary Medicine of the University of Salvador, Bahia. Causes of death could not be determined unambiguously for these animals. All carcasses were maintained at −20°C until complete necropsies were performed. The final sample comprised 112 opossums belonging to five different species (Didelphis aurita, Didelphis albiventris, Marmosops incanus, Micoureus demerarae, and Metachirus nudicaudatus) (Table 1).

**TABLE 1:** Sample characteristics

Species	*n*	No. of positive samples/total no. of samples (%)^*a*^		
		Blood		Liver
		PCR	Serology	PCR
Didelphis aurita	96	0/45 (0)	12/45 (26.6)	1/51 (1.96)
Didelphis albiventris	2	0/1 (0)	0/1 (0)	0/1 (0)
Didelphis sp.^*b*^	4	NT	NT	0/4 (0)
Marmosops incanus	1	NT	NT	0/1 (0)
Micoureus demerarae	7	0/7 (0)	0/7 (0)	NT
Metachirus nudicaudatus	2	0/2 (0)	0/2 (0)	NT
Total	112	0/55 (0)	12/55 (21.8)	1/57 (1.8)

a NT, not tested.

b The species could not be typed unambiguously according to morphological criteria due to potential hybridization events between parental lineages.

### Hepatovirus detection and organ tropism.

Serum and liver specimens were investigated for hepatovirus RNA by nested reverse transcription-PCR (RT-PCR) as described previously (5). Only a single liver specimen from an adult female Brazilian common opossum (Didelphis aurita) termed Br225, originating from Salvador, tested positive. D. aurita was also the most abundantly sampled species in our study (Table 1). The 0.9% rate (1 of 112 animals) of acute hepatovirus infection in opossums, as evidenced by detection of hepatoviral RNA, was not significantly different (Fisher's exact, two-tailed test; *P* = 0.56) from the 0.7% rate (117 of 15,987 animals) found in a large investigation of different small mammals for hepatoviruses (5). This suggested similarities in the epidemiology of hepatovirus infection between marsupials and other small mammals.

**FIG 2:**
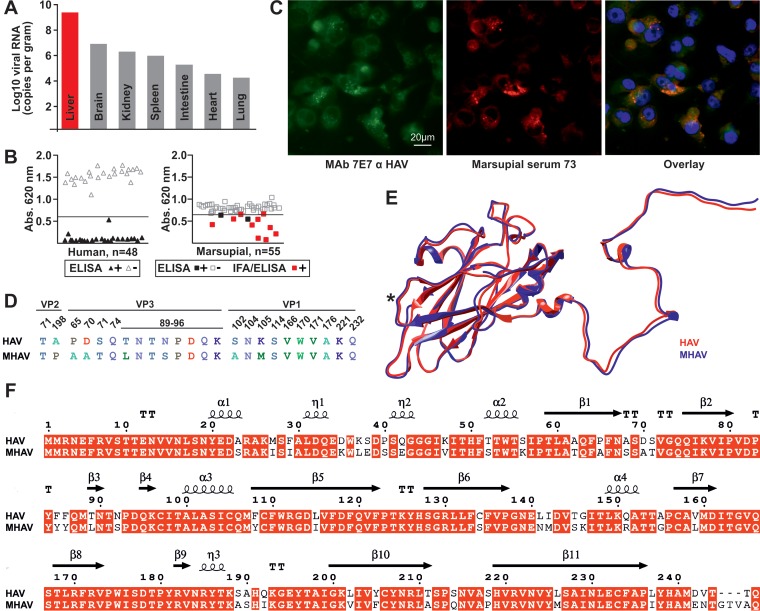
Infection patterns. (A) Marsupial HAV (MHAV) RNA concentrations in solid organs. (B) Adaptation of a human HAV ELISA (triangles) for use on marsupials (squares). Abs., absorbance. (C) Colocalization of antibodies from marsupial serum and an anti-HAV monoclonal antibody (7E7) in FRhK-4 cells infected to 100% with human HAV, mixed with noninfected cells at a 1:1 ratio to generate internal negative controls. (D) Hepatovirus epitopes associated with neutralization, as summarized previously (4). Residue colors indicate biochemical properties according to the Blosum62 matrix. (E) Thermodynamic modeling of MHAV VP3 compared to HAV VP3. *, central antigenic site. (F) Conservation of predicted structural elements within the VP3 domain of HAV and MHAV. Conserved characters are indicated with red boxes; α-helices and 310-helices (η) are represented by squiggles, β-strands are represented by arrows, and strict β-turns are indicated by “TT.”

Following nucleotide sequencing of the screening RT-PCR amplicon, a real-time RT-PCR assay relying on photometrically quantified cRNA standards was designed to determine virus concentrations in different organs of animal Br225 that tested positive for hepatovirus RNA. The hepatovirus RNA concentration in liver tissue was high, at 2.5 × 10^9^ genome copies/g, and was 2 to 5 orders of magnitude higher than those in other solid organs (Fig. 2A). This suggested hepatotropism of the marsupial HAV (MHAV). Whether the deceased animal Br225 suffered from acute hepatitis remained unknown, since the quality of the available tissue specimens was insufficient for histopathological analyses. During macroscopic examination, the animal showed signs of physical injury, including hemorrhages in the liver and lungs. It is thus unlikely that MHAV infection was the direct cause of death in that animal.

### Hepatovirus seroprevalence.

HAV seroprevalence studies in small mammals are challenging due to the lack of established methods. In pivotal investigations of African bats, the HAV seroprevalence obtained using an immunofluorescence assay (IFA) and confirmatory neutralization tests (NTs) was 7.3% (5). Advantages of IFA include the ability to visualize and titrate antibody responses, but sensitivity may be limited. Advantages of NTs include high specificity, but the protocol is laborious and particularly time-consuming for HAV, thus not facilitating high-throughput analyses (14). In preliminary experiments, we determined that results of an HAV enzyme-linked immunosorbent assay (ELISA) relying on competition of serum antibodies with a horseradish peroxidase-conjugated polyclonal human anti-HAV IgG were equivalent to those of NTs for all bat sera tested previously for HAV (5). For 48 human sera used to assess ELISA performance, positive and negative results were clearly distinguishable (Fig. 2B). The mean optical density (OD) for 24 positive sera from HAV vaccinees was 0.114 (standard deviation [SD], 0.122), compared to a mean OD of 1.916 (SD, 0.191) for 24 negative sera. Among opossum sera, negative and positive samples were less clearly distinguished. The mean OD for 12 positive sera was 0.426 (SD, 0.205), compared to a mean OD of 0.807 (SD, 0.068) for 43 negative sera. Nonetheless, the difference in ODs between positive and negative sera was statistically significant for both humans and marsupials (Mann-Whitney test; *P* < 0.001 for both hosts). High specificity of serological detection was also suggested by confirmation of positive ELISA results by use of an IFA relying on cells infected with human HAV for 10 of 12 marsupial specimens and by colocalization of marsupial antibodies with a monoclonal control antiserum raised against human HAV (Fig. 2C). Additionally, high specificity of serological detection was suggested by relatively high endpoint IFA titers (median, 1:900; range, 1:40 to 1:10,000) (Table 2). In sum, we found common exposure of Brazilian common opossums to hepatoviruses, at 26.6% (12 of 45 sera from D. aurita). None of the 10 sera available from other opossum species yielded positive ELISA results (Table 1).

**TABLE 2:** Individual test results^*c*^

Sample ID^*a*^	ELISA OD^*b*^	IFA endpoint titer	PCR result		Sampling site
			Blood	Liver	
8	0.074	1:10,000	neg	NT	Mata de São João
2	0.640	1:1,000	neg	NT	Salvador
67	0.349	1:200	neg	NT	Salvador
70	0.630	neg	neg	NT	Salvador
73	0.206	1:800	neg	NT	Salvador
80	0.544	1:1,000	neg	NT	Salvador
135	0.541	neg	neg	NT	Salvador
157	0.647	1:400	neg	NT	Salvador
158	0.408	1:1,000	neg	NT	Salvador
196	0.650	1:100	neg	NT	Salvador
209	0.535	1:40	neg	NT	Salvador
225	NT	NT	NT	pos	Salvador
233	0.107	1:10,000	neg	NT	Salvador

a All animals belonged to the species D. aurita.

b Cutoff for positivity, ≤0.650; extinction at 620 nm.

c pos, positive; neg, negative; NT, not tested.

During clinical examination considering general condition, nutritional and hydration statuses, heart and respiratory rates, and body temperature, no abnormalities were observed in seropositive opossums. This may suggest that opossums generally survive hepatovirus infection. Seropositive animals originated from both sampling sites, suggesting a relatively wide spread of MHAV, since the home range of Brazilian common opossums is only about 0.012 km^2^ (15).

### Antigenic relatedness between HAV and MHAV.

Marsupial sera reacted with HAV antigens in ELISA and IFA. This suggested either exposure of marsupials to human HAV or antigenic relatedness between HAV and MHAV. To further examine antigenic relatedness between HAV and MHAV, all epitopes associated with HAV neutralization were analyzed. As shown in Fig. 2D, 17 of 24 (77.3%) amino acid residues across VP2, VP3, and VP1 were either identical or homologous between HAV and MHAV. Thermodynamic modeling of MHAV VP3 revealed very high structural similarity between HAV and MHAV, including a conformational epitope constituting the major antigenic site of HAV (shown with an asterisk in Fig. 2E) and all structural elements defining the structure of HAV VP3 (Fig. 2F). In sum, serological reactivity patterns, including high seroprevalence and antibody titers, as well as genomic and structural similarities suggested exposure of animals to MHAV rather than HAV and considerable antigenic relatedness between HAV and MHAV.

### Genomic characterization of the marsupial hepatovirus.

**FIG 3:**
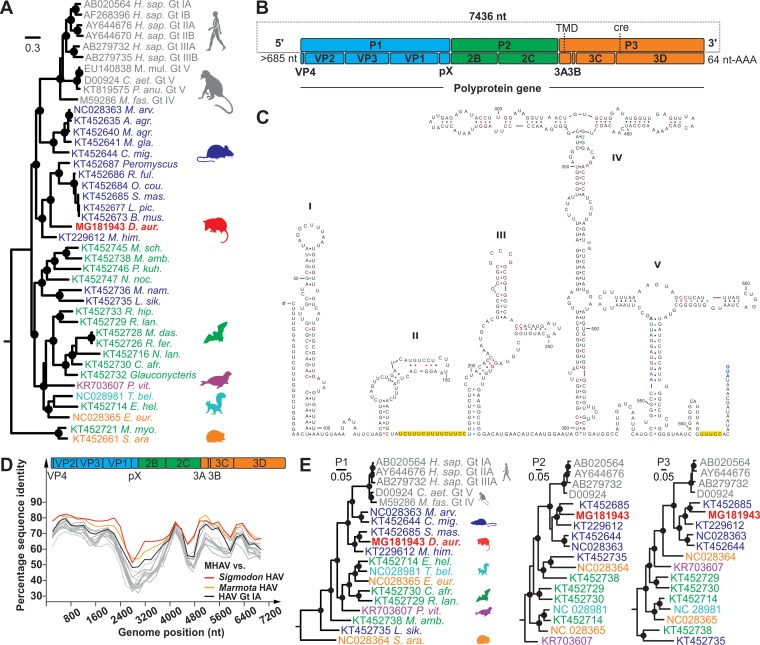
Genomic characterization and evolutionary relationships. (A) Partial VP2-VP3 hepatovirus phylogeny based on 864 nucleotides, corresponding to genomic positions 1,124 to 1,988 in HAV genotype IA (GenBank accession no. AB020564). Gt, genotype. (B) Full-genome organization of marsupial HAV (MHAV). Sequencing of the 5′ terminus likely lacked 33 nucleotides compared to the sequence of the most closely related rodent HAV strain from Sigmodon mascotensis (accession no. KT452685). TMD, transmembrane domain; cre, *cis*-acting replication element. (C) IRES prediction. Yellow, pyrimidine-rich regions. (D) Amino acid sequence identities between MHAV and rodent HAVs from S. mascotensis (red), Marmota himalayana (yellow; accession no. KT229612), and human HAV genotype IA (black). Top, schematic representation of the HAV genome organization. All other hepatoviruses are given in gray for clarity of presentation. (E) Bayesian phylogenies of hepatovirus domains P1, P2 (only 2C), and P3 (only 3CD). Host genera and species are abbreviated for graphical reasons, and full host details are provided in Fig. 6C. Filled circles in panels A and E show Bayesian posterior probabilities of grouping above 0.9. Each scale bar indicates genetic distance, and virus names are colored according to host order.

In a Bayesian phylogeny of the partial VP2-VP3 region obtained upon sequencing of an extended amplicon yielded by the screening PCR assay as described previously (5), MHAV clustered within a clade defined by rodent hepatoviruses from Chinese woodchucks (Marmota himalayana) and Mexican cotton rats (Sigmodon mascotensis) (5, 7), with high statistical support (Fig. 3A). The nearly complete MHAV genome was characterized by sets of nested PCR assays and a 5′/3′ rapid amplification of cDNA ends (RACE) strategy as described previously (5). Similar in length to that of HAV, the MHAV polyprotein gene comprised 6,687 nucleotides and showed the typical properties defining hepatoviruses. This included the absence of a leader protein and the presence of a truncated VP4 protein lacking an N-terminal myristoylation signal, a tandem YPX_3_L late domain motif in VP2 likely involved in quasi-envelope acquisition (16), a predicted transmembrane domain (TMD) in the 3A domain (17), and a *cis*-acting replication element (*cre*) in the 3D^pol^ domain (18) (Fig. 3B). The polyprotein gene was preceded by a 5′ untranslated region containing a predicted type 3 internal ribosomal entry site (IRES) (19), which consisted of five major domains and included typical pyrimidine-rich regions between domains II and III and before the start codon of the polyprotein gene (Fig. 3C). Along the polyprotein gene, MHAV genomic identity was generally highest with a rodent hepatovirus sampled in Mexico in 2005 from a cotton rat (S. mascotensis) (5) (Fig. 3D). Averaged over the translated full polyprotein gene, the amino acid sequence distance between MHAV and the Mexican rodent HAV was 23.7%. For comparison, amino acid sequence distances between MHAV and human HAV genotypes ranged from 32.3 to 32.8%, and the maximum sequence distance within HAV genotypes was 7.9%. This suggested that MHAV is a new Hepatovirus species. As shown in Table 3, predicted polyprotein cleavage sites were highly conserved between HAV and MHAV. This suggested similarities in 3C^pro^-mediated polyprotein processing between HAV and MHAV (20), likely including a C-terminal extension of VP1, termed pX, potentially involved in capsid assembly and quasi-envelopment (16, 21). The close genetic relationship of MHAV with Chinese and Mexican rodent hepatoviruses was confirmed along the major picornavirus domains, P1, P2, and P3, in separate Bayesian phylogenies (Fig. 3E). No evidence for recombination involving MHAV was evident from these phylogenies, and no recombination involving MHAV was detected in formal recombination analyses of the full MHAV genome.

**TABLE 3:** Similarities between predicted polyprotein cleavage sites of MHAV and HAV^*a*^

Virus	Predicted cleavage site								
	P1			P2		P3			
	VP4/VP2	VP2/VP3	VP3/VP1-PX	VP1-px/2B	2B/2C	2C/3A	3A/3B	3B/3C	3C/3D
HAV	**LSLA**/**D**I**EE**	LS**TQ**/**MMRN**	VTT**Q**/V**GDD**	LF**SQ**/AKIS	**L**R**TQ**/S**FS**N	**LWSQ**/GIS**D**	**IPA**E/**GVYH**	V**ESQ**/**STLE**	**IESQ**/**RIMK**
MHAV	**LSLA**/**D**V**EE**	IM**TQ**/**MMRN**	TVA**Q**/A**GDD**	VC**SQ**/SGPI	**L**H**TQ**/G**FS**D	**LWSQ**/SGD**D**	**IPA**C/**GVYH**	A**ESQ**/**STLE**	**IESQ**/**RIMK**

a The HAV reference sequence under accession no. NC_001489 was used. Amino acids in bold indicate conserved residues.

### Codon usage of the novel hepatovirus and marsupial hosts.

**FIG 4:**
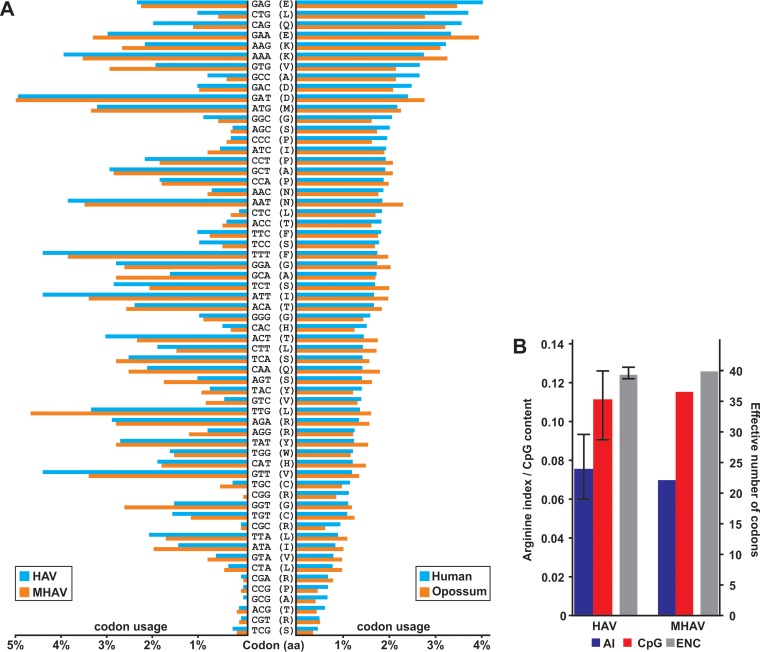
Codon usage bias. (A) Codon usages of HAV and MHAV compared to those of human and marsupial hosts. Stop codons are not shown for clarity of presentation. aa, amino acid residue. (B) AIs, corrected CpG dinucleotide contents, and effective numbers of codons (ENC) of HAV and MHAV. Error bars for HAV indicate the ranges of values among HAV genotypes.

One of the most distinctive features of HAV is a marked codon usage bias (CUB), reflected as a small effective number of codons (ENC) (22). In HAV, CUB has been associated with preferred usage of rare codons to modulate translation and evade host cell defenses (22). As shown in Fig. 4A, codon usages were generally comparable between HAV and MHAV and between human and marsupial hosts. The CUBs of HAV and MHAV were very similar as evidenced by comparable ENC counts and arginine indexes (Fig. 4B). Rather than a preferred usage of codons that were underrepresented in their hosts, HAV and MHAV shared a pronounced avoidance of codons containing a CpG dinucleotide. In many RNA viruses, CUB may largely result from selective pressure against CpG dinucleotides (23), which were recently shown to be targeted by the zinc finger antiviral protein ZAP (24). In HAV and MHAV, the CpG contents were similarly low, at 0.111 and 0.115 of the expected content given random dinucleotide composition at the actual frequencies of genomic C and G nucleotides, much lower than the CpG content observed in other picornaviruses (25). Therefore, it is likely that HAV and MHAV are under similar selective pressure, hypothetically mediated by ZAP.

### Evolutionary relationships of the novel marsupial hepatovirus.

The level of congruence between host and virus phylogenies can provide information on the genealogy of extant viruses. Programs for coevolutionary analyses can be summarized broadly into two groups (26). The first group of programs aims at testing the congruence between host and virus phylogenies by identifying symmetries in genetic distances. In the case of this study, we used a program termed ParaFit (27). The second group of programs aims at comparing the topologies of host and virus phylogenies to infer the nature and frequency of different evolutionary events. In the case of this study, we used a program termed CoRe-PA (28). Both ParaFit and CoRe-PA were used for two different Hepatovirus data sets. The first Hepatovirus data set consisted of the partial VP2-VP3 domains. This data set was chosen because it contains the largest number of novel hepatovirus sequences from diverse hosts (5). The second Hepatovirus data set consisted of the full P1, P2, and P3 polyprotein gene domains. This data set was chosen because evolutionary associations can differ between different domains of picornavirus polyprotein genes due to recombination events (29). The host phylogeny was reconstructed using a mitochondrial marker and taxonomic constraints as detailed previously (5) to increase the phylogenetic resolution to above the family level.

**FIG 5:**
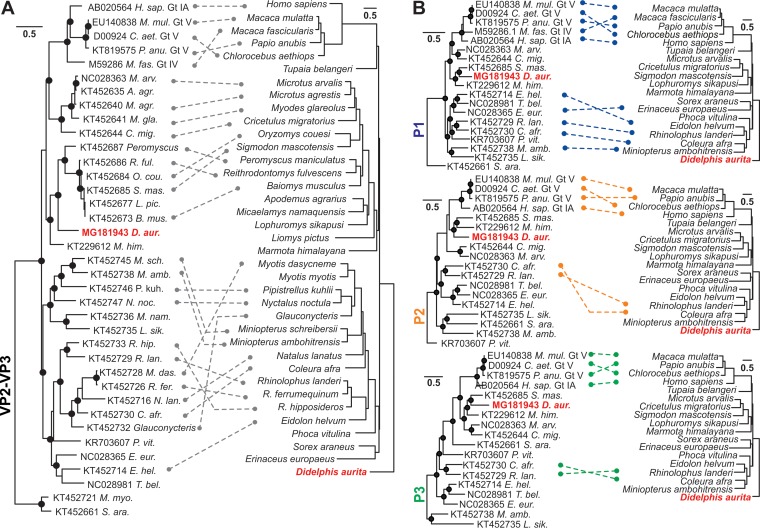
Distance-based coevolutionary analyses. (A) Partial VP2-VP3 domains. Dashed lines, significant (*P* < 0.05) coevolutionary associations between individual hosts and hepatoviruses. Filled circles, Bayesian posterior probabilities of grouping above 0.9. The scale bar indicates genetic distance. (B) Hepatovirus domains P1, P2, and P3, represented as described for panel A. The novel MHAV is highlighted in bold red.

Using ParaFit, the overall agreement between virus and host phylogenies was highly significant for the analyses of both the partial Hepatovirus VP2-VP3 domains and the full polyprotein domains (*P* < 0.01). Striking examples of cosegregating hepatoviruses and bat hosts from geographically distant sampling sites included closely related viruses from African and European Miniopterus and Rhinolophus bats (Fig. 5A). In contrast, fewer nonprimate hepatoviruses and their hosts yielded statistical significance in analyses of the full polyprotein domains (Fig. 5B). Consistent with the results for the partial VP2-VP3 data set, most of the statistically significant individual associations were observed in the capsid-encoding P1 domain that includes the VP2-VP3 domains.

Despite the statistical significance of the distance-based analyses, the topology of the hepatovirus phylogenies did not match that of their hosts on numerous occasions. Additionally, nearly identical SHAV genotype V genomic sequences are available from three distinct genera of nonhuman primates, namely, Papio anubis (olive baboons), Macaca mulatta (rhesus macaques), and Chlorocebus aethiops (African green monkeys) (6, 30, 31). Similarly, nearly identical nonprimate hepatoviruses are available from sympatric yet genetically distinct rodent and bat hosts (Fig. 5A). The identification of the natural hosts for these hepatoviruses is thus challenging, and recent spillover infections facilitated by sympatry and host genetic relatedness (12) may bias the distance-based coevolutionary reconstructions.

**FIG 6:**
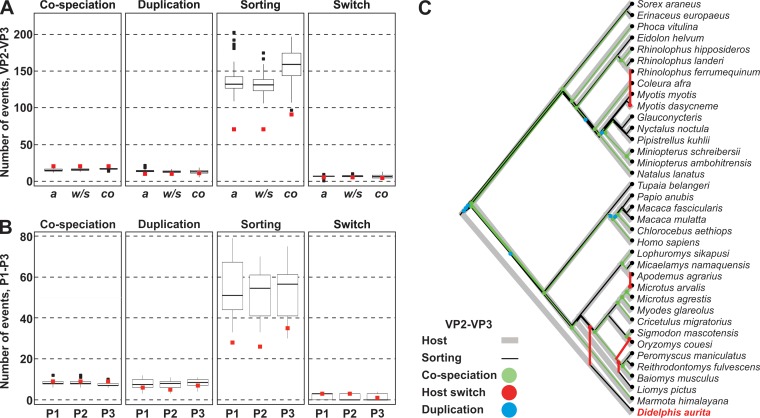
Event-based coevolutionary analyses. (A) Frequencies of evolutionary events (indicated above panels) in the partial VP2-VP3 data set. Red squares, original data set. Tukey box plots show data from 100 randomizations of host-virus associations. *a*, automated cost model; *w*/*s*, automated model excluding reconstructions without host switches; *co*, reconstructions facilitating cospeciations by low event costs. (B) Frequencies of evolutionary events, as described for panel A, for P1, P2, and P3 domains. Confirmatory runs using cost models excluding reconstructions without host switches and those maximizing cospeciation yielded nearly identical results and are not shown for clarity of presentation. (C) Representation of the most parsimonious reconciliation of host and hepatovirus phylogenies for the partial VP2-VP3 domains.

Event-based reconciliations of hepatovirus and host phylogenies by use of CoRe-PA revealed that, indeed, 17 to 21% of evolutionary events were projected to cospeciations for the partial VP2-VP3 domains (Fig. 6A, left panel) and the P1, P2, and P3 domains (Fig. 6B, left panel). The existence of coevolutionary events was consistent with fewer cospeciation events in the majority of control runs relying on randomized host-virus associations than those in the original data sets (red squares in Fig. 6A and B). A similar quantity of cospeciation events in the VP2-VP3 data set was also observed in control runs which excluded all reconstructions without at least one host switch (abbreviated “*w/s*” in Fig. 6A) or which facilitated reconstructions of cospeciations by low costs attributed to those events (abbreviated “*co*” in Fig. 6A). However, host-independent evolutionary events predominated the genealogy of extant hepatoviruses. As shown in Fig. 6A and B, these events included predominantly sorting events, i.e., viruses were predicted to have remained in only one host progeny lineage after speciation. This may result either from failure of the virus to speciate along with the host or from extinction of one virus progeny lineage. Hypothetically, sorting events may also represent unrecognized cospeciations due to lack of sampling of the respective hepatovirus progeny counterparts. Additional host-independent events included duplications, i.e., host-independent viral speciation events, and host switches. As shown in Fig. 6C, the phylogenetic relationship of MHAV was reconstructed as the most deep-branching host switch among extant hepatoviruses, highlighting the relevance of including MHAV in Hepatovirus evolutionary reconstructions.

Common challenges of coevolutionary analyses include partially unresolved virus phylogenies and an inevitable sampling bias, which we tried to minimize by multiple levels of bioinformatic analysis. In sum, distance-based and event-based coevolutionary reconstructions yielded limited evidence for cospeciation of hepatoviruses and their hosts, whereas event-based reconstructions revealed that host-independent evolutionary events predominated the genealogy of extant hepatoviruses. Despite our thorough cophylogenetic analyses, partially contradictory results suggest that the macroevolutionary history of hepatoviruses is too complex to be described exhaustively using the currently available tools. Future analyses would greatly benefit from event-based tools taking branch lengths into account and tools recognizing more than just four evolutionary events (32).

## DISCUSSION

We identified a new hepatovirus in marsupials and investigated its prevalence and organ distribution. Marsupials such as opossums are well-known reservoirs of human-pathogenic parasites, such as Leishmania and Trypanosoma (33, 34). In contrast, little is known about viruses in opossums, despite the increased attention small mammals gained as evolutionary sources of major human viruses, including HAV (5). Limited genomic data from opossums have been retrieved for hantaviruses, anelloviruses, orthopoxviruses, flaviviruses, and parvoviruses (35–39). The broad taxonomic range of viral findings may suggest an important role of opossums as sources of human infections, because many American marsupial species are synanthropic (40). Additionally, opossums are commonly hunted and consumed as wild game by resource-limited Brazilian communities (41). Because the Hepatovirus clade containing MHAV shares a monophyletic ancestor with human HAV, MHAV may hypothetically retain the ability to infect humans. However, the conserved antigenicity between HAV and MHAV may limit an introduction of MHAV or related viruses into humans due to community protective immunity to HAV. Notably, community protective immunity may be elicited both by frequent natural infection with HAV, suggested by the HAV seroprevalence of about 70% in young adults in northeastern Brazil (42), and by vaccination programs initiated in Brazil in 2014 (43).

Our formal coevolutionary analyses confirmed a complex genealogy of the genus Hepatovirus. We obtained statistically significant evidence for the existence of cosegregation of distinct hepatoviruses and their hosts separated by large geographic distances, as suggested before for some bat hepatoviruses (6). However, event-based reconstructions suggested that host-independent macroevolutionary patterns predominate the genealogy of extant hepatoviruses. Predominantly host-independent Hepatovirus evolution is consistent with a lack of coevolutionary relationships in other Picornaviridae genera (44, 45). Irrespective of the inherent uncertainties of coevolutionary reconstructions, host switches, such as that from putative rodent-borne MHAV ancestors into marsupials, highlight the fact that hepatoviruses can infect highly diverse hosts. The inferred cross-order hepatovirus host switch from rodents into marsupials may have been facilitated by sympatry of these hosts in many areas of Latin America, including competition for food and even marsupials feeding on rodents (46, 47).

Our results strongly suggest that the species barriers toward hepatovirus infection seem penetrable, hinting at a broadly conserved cellular receptor across mammalian hosts, which may extend beyond the canonical HAV receptor, HAVcr1/TIM-1, according to recent data (48). Immune control of HAV includes components of the innate and the adaptive immune response. Regarding innate immune responses, cleavage of mitochondrial antiviral signaling protein (MAVS) is a major marker of HAV immune evasion and host specificity (49). Comparative investigation of MAVS cleavage by MHAV protease precursors may thus yield further insights into hepatovirus species barriers. Regarding adaptive immune responses, it is noteworthy that major components of the adaptive immune system likely evolved before the divergence of marsupials from Eutherians (50). Nonetheless, marsupial CD4 sequences share only about 40% amino acid identity with those of Eutherian mammals (51). Because immune control of HAV includes antiviral cytokines excreted by CD4^+^ T cells (52), MHAV may represent a unique opportunity to assess whether this part of the immune control of HAV is indeed evolutionarily conserved, once virus isolates or a reverse genetics system becomes available. Finally, opossums have been used previously in studies of viral pathogenesis and transmission, including studies of rabies virus, vesicular stomatitis virus, and La Crosse virus (53–55), highlighting the feasibility of experimental hepatovirus infections in these uniquely divergent animals.

## MATERIALS AND METHODS

### Ethical aspects.

Sampling and export of specimens were approved by the Ethics Committee on Animal Use of the School of Veterinary Medicine and Animal Science of the Federal University of Bahia (permit number 25/2014), by the System of Authorization and Information on Biodiversity (SISBio/ICMBio) (permit numbers 43737-2 and 43737-4), by the Ministry of Agriculture, Livestock and Food Supply (MAPA) (permit numbers 005/2014 and 001/2017), and by CITES permits E-00306/17 (Germany) and 15BR018932/DF and 16BR022344/DF (Brazil).

### Sampling.

Animals were captured using tomahawk traps with fruit and meat as bait. After sedation by trained veterinarians, blood was collected from cephalic, lateral saphenous, jugular, femoral, or caudal veins, depending on the species and size of the captured animal. Animals were subsequently released. Tissues from animals obtained dead were stored in RNAlater solution (Qiagen, Hilden, Germany) after necropsy. Animals were typed according to morphological criteria by trained veterinarians.

### RNA purification.

Viral RNA was extracted from about 30 mg of tissue from solid organs or from 10 to 50 μl of serum. RNA was purified using the MagNA Pure 96 DNA and Viral NA large-volume kit (Roche, Penzberg, Germany) for tissue specimens and the DNA and Viral NA small-volume kit (Roche) for sera.

### Hepatovirus detection and quantification.

Viral quantification was done by strain-specific real-time RT-PCR, using *in vitro*-transcribed cRNA control standards, as described previously (56). Primers and probes for this assay were targeted to the VP2 domain and contained the following sequences: MHAV-rtF, AATCCTACACCCTTTCAACAAGGA (polyprotein gene positions 409 to 432); MHAV-rtR, AGGGTATACAGGCAAAGAAGCAA (polyprotein gene positions 479 to 501); and MHAV-rtP, 6-carboxyfluorescein (FAM)-TTGCAGCAATGGTCCCAGCAGATC-6-carboxytetramethylrhodamine (TAMRA) (polyprotein gene positions 440 to 463). Thermocycling involved reverse transcription at 55°C for 20 min followed by 94°C for 3 min and then 45 cycles of 94°C for 15 s and 58°C for 30 s. PCR chemistry was generally performed by use of the OneStep SuperScript III RT-PCR kit for first-round reactions and the Platinum *Taq* PCR kit (both from Thermo Fisher, Darmstadt, Germany) for second-round reactions, as described previously (5).

### Serology.

An ELISA system with increased sensitivity for the detection of antibodies to human HAV was used for antibody detection in marsupial sera, following the manufacturer's specifications (Mediagnost, Reutlingen, Germany). The ELISA is based on the competition of serum antibodies with a high-avidity horseradish peroxidase-conjugated polyclonal human anti-HAV IgG for detection. Sera were tested at a 1:20 dilution. Due to limited serum volumes, only one replicate was tested in single determinations. Negative controls included ELISA dilution buffer and anti-HAV-negative human sera. The cutoff was defined as 50% inhibition of the extinction of the negative controls.

An indirect IFA was done using FRhK-4 cells persistently infected with HAV as described previously (5). Opossum sera were diluted 1:40, 1:100, 1:200, 1:400, 1:800, 1:1,000, 1:10,000, and 1:20,000. Reactions were detected by use of rabbit anti-opossum IgG (Biomol, Hamburg, Germany) and a cyanine 3-labeled goat anti-rabbit IgG (Dianova, Hamburg, Germany). Infected cells were mixed with noninfected cells at a 1:1 ratio to allow for internal negative controls. Additionally, mock-infected cell cultures were used as controls with opossum sera to exclude nonspecific reactivity. Both the ELISA and IFA thus did not differentiate between IgM and IgG isotypes in analyzed sera.

### Bioinformatics.

Genome annotations and translation alignments were done using Geneious 6.1.8 (57). Nexus files for phylogenetic analyses were generated in MEGA7 (58). Bayesian phylogenies were generated using MrBayes V3.1, using a GTR+G+I substitution model (59). Trees were run for 2 million generations and sampled every 100 steps. After exclusion of 5,000 of the total of 20,000 trees as burn-in, final trees were annotated by use of TreeAnnotator and visualized with FigTree from the BEAST package (60). Avian encephalomyelitis virus (genus Tremovirus) was used as an outgroup. Secondary structure predictions were done using Mfold (61). Sequence distances were plotted using SSE V1.2 (62). The codon usage and relative CpG content of viruses were determined using SSE V1.2 (62). The arginine index (AI) was calculated as the ratio of genomic CGN/(CGN + AGR) codons. Host codon usage was retrieved from the online database HIVE-CUT (63). Opossums were represented by Monodelphis domestica in HIVE-CUT. Host cytochrome B sequences were obtained from GenBank and used for reconstruction of phylogenetic relationships as described above. ParaFit (27) was run in R (V3.4.1) through the Rstudio environment (V1.0.153), with the packages APE (V4.1) and Vegan (V2.4-3), with 100,000 random permutations of virus-host associations included to test for statistical significance. Most parsimonious reconciliations between hepatovirus and host phylogenies were computed with the command line version of CoRe-PA (28), using three different event cost evaluation methods for distinct evolutionary events, namely, cospeciation, duplication, sorting, and host switching. First, the adaptive cost method implemented in CoRe-PA was used with 1,000 random cost models. Second, the adaptive cost method was used as before, but only reconciliations with at least one host switch (-s option in CoRe-PA) were considered. Third, cospeciation events were facilitated in reconciliations by low event costs (-x option in CoRe-PA). Host-virus associations were randomized to yield 100 replicates, and the results were compared with the reconciliation obtained from the original data set. Thermodynamic modeling of MHAV VP3 was done on the HAV crystal structure (4) by use of Chimera and ESPript 3.0 (64, 65).

### Accession number(s).

The MHAV genome was submitted to GenBank under accession number MG181943.
